# Micro-CT X-ray imaging exposes structured diffusion barriers within biofilms

**DOI:** 10.1038/s41522-018-0051-8

**Published:** 2018-04-17

**Authors:** Alona Keren-Paz, Vlad Brumfeld, Yaara Oppenheimer-Shaanan, Ilana Kolodkin-Gal

**Affiliations:** 1 Department of Molecular Genetics, Weizmann Institute of Science, 76100 Rehovot, Israel; 2 Chemical Research Support, Weizmann Institute of Science, 76100 Rehovot, Israel

## Abstract

In nature, bacteria predominantly exist as highly structured biofilms, which are held together by extracellular polymeric substance and protect their residents from environmental insults, such as antibiotics. The mechanisms supporting this phenotypic resistance are poorly understood. Recently, we identified a new mechanism maintaining biofilms - an active production of calcite minerals. In this work, a high-resolution and robust µCT technique is used to study the mineralized areas within intact bacterial biofilms. µCT is a vital tool for visualizing bacterial communities that can provide insights into the relationship between bacterial biofilm structure and function. Our results imply that dense and structured calcium carbonate lamina forms a diffusion barrier sheltering the inner cell mass of the biofilm colony. Therefore, µCT can be employed in clinical settings to predict the permeability of the biofilms. It is demonstrated that chemical interference with urease, a key enzyme in biomineralization, inhibits the assembly of complex bacterial structures, prevents the formation of mineral diffusion barriers and increases biofilm permeability. Therefore, biomineralization enzymes emerge as novel therapeutic targets for highly resistant infections.

## Introduction

Historically, bacteria were viewed as unicellular organisms that struggle for individual survival. However, it is now clear that in nature bacteria predominantly exist as biofilms — complex differentiated communities held together by an extracellular polymeric substance. In the biofilm, individual cells take part in complex multi-cellular processes using a variety of chemical and metabolic cues to coordinate activity within the community, as well as across species.^1–3^ In the past few decades, it became clear that biofilms play a prominent role in disease, due to their increased antibiotic resistance as compared with planktonic cells. Biofilms formed by septic pathogens contribute to morbidity and mortality of patients with implants, chronic/burn wounds and cancer.^4,5^

Organic extracellular polymeric substance is composed of polysaccharides, proteins, nucleic acids, lipids and other biological macromolecules, and its’ production has been extensively studied as a means of cell-cell and cell-substrate adhesion.^3,6,7^ Recently, we and others showed that precipitation of calcium carbonate contributes to the assembly of the complex biofilm architecture.^8–13^ While calcium is available from the environment, bicarbonate is actively produced by CO_2_ hydration (CO_2_ + H_2_O ↔ HCO_3_ + H^+^), where the source of CO_2_ can be a byproduct of bacterial metabolism or of the immediate environment.^14–16^ In clinic, precipitation of calcium carbonate was shown to promote catheter colonization, active infection and interspecies interactions.^8,10,11^ In this context it is important to note that diffusion of simple small molecules in calcite is reduced by several orders of magnitude compared with their diffusion in a polysaccharide gels and fibers,^17–20^ and this is very likely to be true also for antibiotics. Therefore, development of sensitive and accurate methods to measure 3D distribution of calcium carbonate in biofilms in their natural setting is of high importance, and may allow to predict the efficacy of antibiotic penetration and the success of antibiotic treatment. Here we propose a novel use for the µCT imaging technique to study the calcium carbonated areas within biofilms and their effect on diffusion of small molecule weight solute.

## Results

In this work, we utilize the fact that µCT allows to obtain complete 3D information on opaque samples, and study the detailed inner structure of calcium minerals within biofilm colonies. As this technique detects structured calcium (such as aggregates and crystals), but not amorphous calcium or calcium salts with organic substances,^21^ it is a useful tool for identifying and analyzing calcium deposits. We show that high resolution µCT provides structural insight into the calcium structures present within the biofilm.

To create the 3D reconstruction, a bacterial colony was grown on biofilm-inducing agar medium for indicated time. The whole, unfixed colony was transferred to a plastic slide, and rotated between the X-ray source and the detector positioned at optimal distances for a cubic voxel size of 0.87 μm. The drying of the sample under X-ray was prevented by mounting the plate in a sealed cell under saturated water vapors atmosphere. 2D projections were taken at different angles over 180^°^. The full set of images was then used to reconstruct the whole volume of the sample by back projection algorithm and thus a high-resolution 3D image was generated (see Sup. Movies 1, 2, 3, 4).

**Fig. 1:**
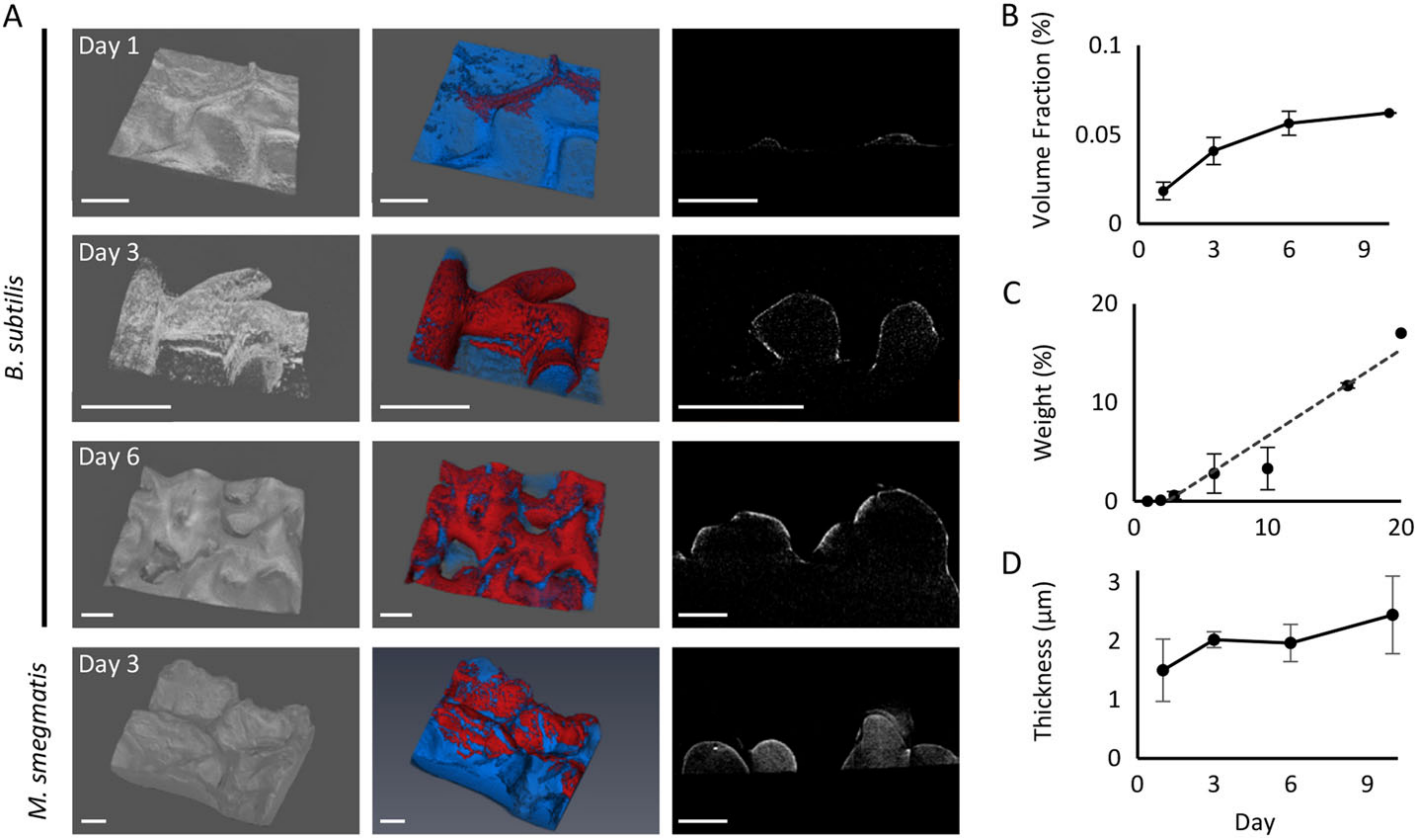
Imaging and quantifying calcium-rich structures in *B. subtilis* colonies. *B*. *subtilis* and *M. smegmatis* colonies grown on 1.5% B4 agar, supplemented with Ca acetate, as indicated in the main text. **a** Left panel—3D reconstruction. Middle panel—segmentation of the reconstructed volume, red indicates the densest mineral. Right panel—transversal slices. Scale bar: 200 µm. **b** Relative volume of the mineral layer of the total *B. subtilis* colony volume. Averages of three independent experiments are displayed, and the bars represent standard deviation. **c** TGA analysis of calcium minerals in *B. subtilis* colonies. Averages of three independent experiments are displayed, and the bars represent standard deviation. **d** Estimated thickness of the mineral layer in *B. subtilis* colony. Averages of three independent experiments are displayed, and the bars represent standard deviation

First, we examined the development of calcium-rich structures in *B*. *subtilis* colonies grown on biomineralization-promoting medium containing 0.25% calcium acetate. 3D reconstruction revealed intricate macro-scale structures (Fig. 1a, left panel). In our previous paper,^12^ we have shown that regions of dense calcium are correlated with the biofilm wrinkles. The observed structures were highly reminiscent of the biofilm wrinkles in time of development, form and location (Fig. 1).

Biomineralization is a universal property of biofilms formed by very different bacterial taxa. To determine whether our observations reflect general architectural principles, we also examined the actinobacterium *Mycobacterium smegmatis*, which is known to form very robust biofilms.^22^ As expected, when the calcium concentration in the growth medium was 0.25% as with *B. subtilis*, the colony accumulated mineral crystals in a calcium-dependent manner making a high-resolution micro-CT X-ray imaging impossible. Lowering calcium concentration by ten-fold allowed us to obtain high-resolution images. Even at low calcium concentration, *M. smegmatis* formed robust and complex calcium-rich structures (Fig. 1a, left panel), consistent with dense and complicated wrinkles.

High-resolution images we obtained allowed us to segment the reconstructed volume, in order to identify and measure calcium in different regions of the colony (Fig. 1a, middle panel). The calcium layer spread over time, and while at day 1 it was mostly observed at the wrinkles, at day 6 most of the colony was covered. Furthermore, we estimated the total volume of the colony and of the calcium layer, and the fraction of the calcium out of the whole colony increased over time (Fig. 1b). This was confirmed by Thermogravimetric analysis, which showed accumulation of calcium in the biofilm colonies over time (Fig. 1c).

Next, virtual transversal slices were generated to investigate the calcium distribution within the sample (Fig. 1a, right panel). The most dense calcium carbonate areas formed crust-like cover over the wrinkles. Using the parallel plate model we calculated the thickness of this layer—approximately 1 µm for *B. subtilis* and 3 µm for *M. smegmatis*. Interestingly, we found that the thickness of this calcium layer was constant throughout the colony and did not increase during *B. subtilis* colony development (Fig. 1d). Instead, during biofilm growth the calcium layer spread and covered more and more of the colony wrinkles (see also Fig. 1a, middle panel).

**Fig. 2:**
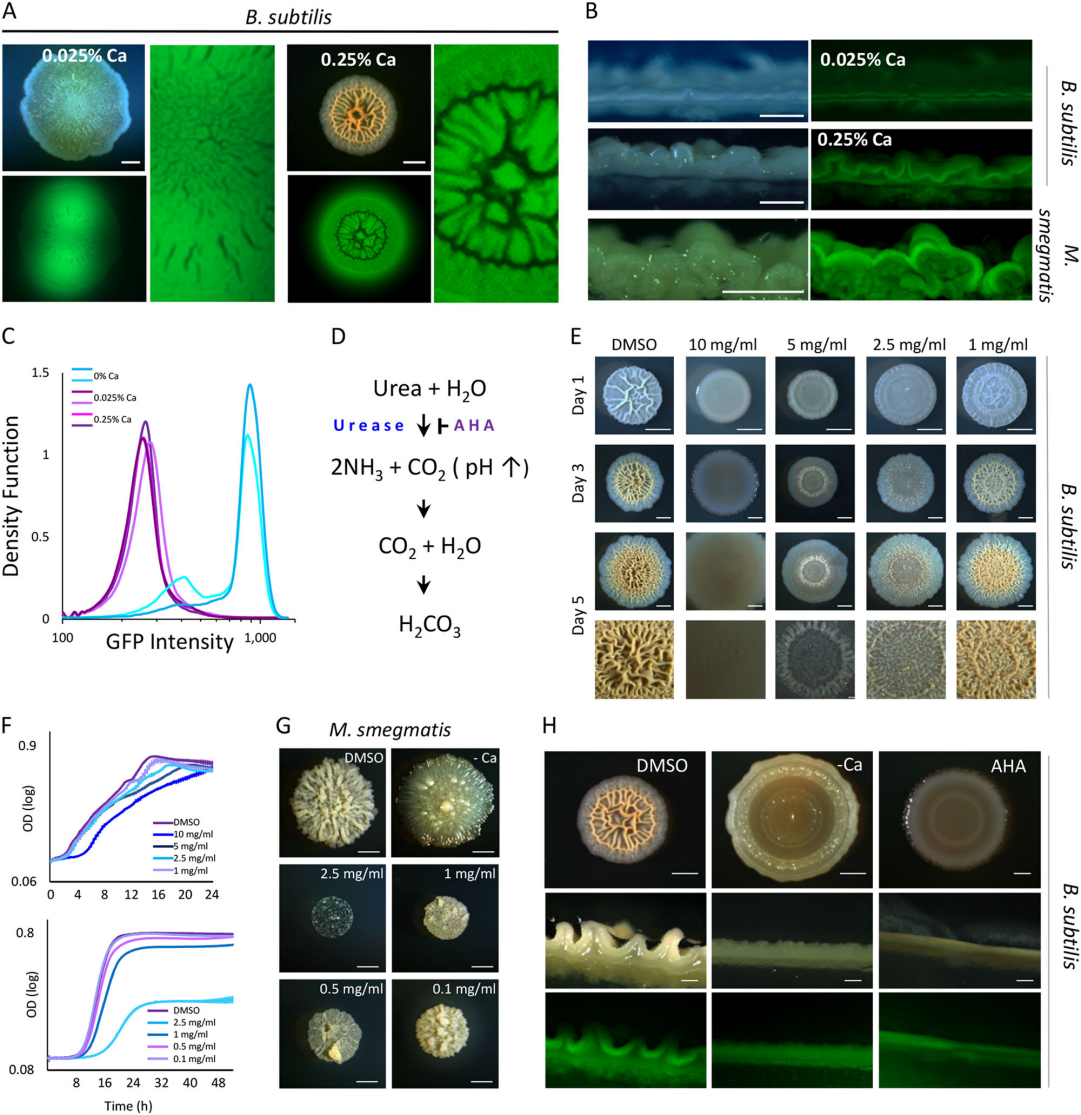
Diffusion through biofilm colonies is limited by calcium-dependent barriers*. B*. *subtilis* and *M. smegmatis* colonies grown on 1.5% B4 agar, supplemented with Ca acetate as indicated. **a** FITC diffusion in *B. subtilis* biofilm colonies. Upper left—bright field, lower left—GFP, right panel—enlargement of lower left image. Scale bars: 2 mm. **b** Cross-sections of colonies taken 4 h after FITC dye was applied. Scale bars: 0.5 mm. **c** FACS analysis of cells stained with CalceinAM. Shown are two independent repeats per treatment. **d** Biomineralization reactions leading to bicarbonate production. AHA acetohydroxamic acid. **e** *B. subtilis* colonies grown on 0.25% Ca, supplemented with AHA as indicated. Scale bars: 2 mm. **f** Planktonic growth in B4 medium, supplemented with Ca and AHA at indicated concentrations. Error bars represent standard deviation. **g** *M. smegmatis* colonies grown either without or in the presence of 0.025% Ca, and AHA at indicated concentrations. Scale bars: 2 mm. **h** Day 3 *B. subtilis* colonies and cross-sections of colonies 4 h after FITC dye was applied. Colonies were grown either on 0.25% or without Ca acetate (−Ca), AHA concentration — 10 mg/ml. Scale bars: upper panel — 2 mm, lower panels — 0.5 mm

While the rigid mineral layer is a structural element, potentially increasing the weight bearing of the wrinkles and supporting the overall colony structure, it might have additional functions. Diffusion of water and small molecular weight solutes is several orders of magnitudes less efficient in calcite^18,19^ compared with organic polymers.^20,23^ Therefore, we hypothesized that calcium carbonate dense areas could function as diffusion barriers. We evaluated the diffusion of water-soluble fluorescein throughout *B. subtilis* biofilm colonies, grown in the presence of high (0.25%) and low (0.025%) levels of calcium. We found that diffusion of fluorescein was limited in the wrinkled biofilm colonies grown in the presence of high calcium concentration. On the other hand, the dye diffused freely in non-wrinkled colonies formed on low calcium concentration (Fig. 2a). In order to gain a better understanding of the causes of this limitation, the colony was manually sliced and the cross-sections were visualized. In colonies grown in the presence of calcium, the diffusion was limited, and the dye accumulated in a discreet area within the colony biomass. This barrier was calcium dependent, and at low calcium concentrations diffusion was less restricted (Fig. 2b). As before, clear diffusion barriers and dye accumulation were also evident in *M. smegmatis*, as would be expected due to its high efficiency in forming biominerals (Fig. 2b). Interestingly, when we determined the intercellular calcium levels by staining cells with calcein AM fluorescent dye, we detected significantly lower levels of *intracellular* calcium in cells grown at higher calcium concentration (Fig. 2c). This lack of correlation between calcium-dependent morphology and intracellular calcium levels suggests that calcium precipitation occurs in a near proximity, but outside the cells, and is consistent with the current view of calcite biomineralization initiation on the outside of the bacterial membrane.^24^

Biomineralization associated with microbial metabolism is usually accompanied by increase in environmental alkalinity, which promotes calcium carbonate precipitation.^14^ One of the central reaction leading to the increased pH is catalyzed by the enzyme urease (Fig. 2d).^25^ The microbial ureases hydrolyze urea to produce carbonate and ammonia, simultaneously increasing the pH and the carbonate concentration, which then combines with environmental calcium to precipitate as calcium carbonate.^25^ Deletion of the *ureA-C* operon prevents biomineralization and the development of complex colony morphology and floating pellicle (Sup. Fig 1,^12^). This effect is very similar to that of low calcium levels in the medium. However, in clinical settings, neither depletion of calcium nor genetic manipulation of multi-species pathogenic biofilms are feasible. Therefore, we tested whether chemically inhibiting urease would lead to inhibition of biomineralization. Inhibition of urease activity by urease inhibitor acetohydroxamic acid (AHA) lead to smooth colony morphology, even in the presence of calcium (Fig. 2e), at concentrations having little or no effect on planktonic growth (Fig. 2f, upper panel). In agreement with our hypothesis that biomineralization mechanisms are shared by different taxa, inhibition of urease activity in *M. smegmatis* inhibited growth, biofilm development and biomineralization. *M. smegmatis* was more sensitive to urease inhibition, suggesting a central role for urease in the physiology of this bacterium, which would be consistent with its higher rates of biomineralization. However, even AHA concentrations which were well-tolerated by planktonic cells (Fig. 2f, lower panel), led to less wrinkled colonies, similar to the effect we achieved by lowering calcium concentration in the growth medium (Fig. 2g). Similar effect on morphology was observed using a pellicle biofilm model system (Sup Fig. 2). Moreover, inhibition of urease decreased non-soluble mineral production (Sup. Fig. 3). Finally, cross-sections of colonies revealed that inhibition of urease prevents the formation of diffusion barriers within the colony (Fig. 2h). Taken together, our results show that chemical inhibition of urease prevents biomineralization and the formation of protective diffusion barriers.

## Conclusions

Bacteria within biofilm communities are up to three orders of magnitudes more resistant to antimicrobial agents and the immune system than planktonic bacteria. Here, we use a µCT X-ray to show that one mechanism for this resistance is the formation of extracellular calcium carbonate sheets that serve as diffusion barriers protecting the colony. Urease is a key enzyme in biomineralization pathway, and chemical inhibition of urease prevents the formation of those diffusion barriers. Thus, urease and other biomineralization enzymes emerge as compelling candidates for the development of novel antibiotics. By using X-ray technologies to image biofilms in medically-relevant settings, it may be possible to predict antibiotic diffusion within biofilms. As the biomineralization is a conserved and wide-spread phenomenon in the bacterial kingdom, it can be potentially targeted to deal with infections resulting from multiple-species, antibiotic-resistant biofilms.

## Electronic supplementary material

Supporting Material

Supporting Movie 1

Supporting Movie 2

Supporting Movie 3

Supporting Movie 4

## References

[1] Stoodley P, Sauer K, Davies DG, Costerton JW (2002). Biofilms as complex differentiated communities. Annu. Rev. Microbiol..

[2] Miller MB, Bassler BL (2001). Quorum sensing in bacteria. Annu. Rev. Microbiol..

[3] Kolter R, Greenberg EP (2006). Microbial sciences: the superficial life of microbes. Nature.

[4] Costerton JW, Stewart PS, Greenberg EP (1999). Bacterial biofilms: a common cause of persistent infections. Science.

[5] Bryers JD (2008). Medical biofilms. Biotechnol. Bioeng..

[6] Branda SS, Vik S, Friedman L, Kolter R (2005). Biofilms: the matrix revisited. Trends Microbiol..

[7] Branda SS, Gonzalez-Pastor JE, Ben-Yehuda S, Losick R, Kolter R (2001). Fruiting body formation by Bacillus subtilis. Proc. Natl. Acad. Sci. USA.

[8] Li X (2015). Spatial patterns of carbonate biomineralization in biofilms. Appl. Environ. Microbiol..

[9] Li X (2016). In situ biomineralization and particle deposition distinctively mediate biofilm susceptibility to chlorine. Appl. Environ. Microbiol..

[10] Li, X., Lu, N., Brady, H. R. & Packman, A. I. Biomineralization strongly modulates the formation of Proteus mirabilis and Pseudomonas aeruginosa dual-species biofilms. *FEMS Microbiol. Ecol.*10.1093/femsec/fiw189 (2016). 10.1093/femsec/fiw189 27697892

[11] Li X, Lu N, Brady HR, Packman AI (2016). Ureolytic biomineralization reduces proteus mirabilis biofilm susceptibility to Ciprofloxacin. Antimicrob. Agents Chemother..

[12] Oppenheimer-Shaanan Y (2016). Spatio-temporal assembly of functional mineral scaffolds within microbial biofilms. NPJ Biofilms Microbiomes.

[13] Dade-Robertson M, Keren-Paz A, Zhang M, Kolodkin-Gal I (2017). Architects of nature: growing buildings with bacterial biofilms. Microb. Biotechnol..

[14] Dhami NK, Reddy MS, Mukherjee A (2013). Biomineralization of calcium carbonates and their engineered applications: a review. Front. Microbiol..

[15] Perito B, Mastromei G (2011). Molecular basis of bacterial calcium carbonate precipitation. Prog. Mol. Subcell. Biol..

[16] Dupraz C (2009). Processes of carbonate precipitation in modern microbial mats. Earth Sci. Rev..

[17] Ibrahim M, Issa M (2016). Evaluation of chloride and water penetration in concrete with cement containing limestone and IPA. Constr. Build. Mater..

[18] Fisler DK, Cygan RT (1998). Cation diffusion in calcite: determining closure temperatures and the thermal history for the Allan Hills 84001 meteorite. Meteorit. Planet Sci..

[19] Fisler DK, Cygan RT (1999). Diffusion of Ca and Mg in calcite. Am. Mineral..

[20] Li TQ, Henriksson U, Klason T, Odberg L (1992). Water diffusion in wood pulp cellulose fibers studied by means of the pulsed gradient spin-echo method. J. Colloid Interf. Sci..

[21] Wang QQ, Zhang CF, Chu CH, Zhu XF (2012). Prevalence of Enterococcus faecalis in saliva and filled root canals of teeth associated with apical periodontitis. Int. J. Oral Sci..

[22] Purdy GE, Pacheco S, Turk J, Hsu FF (2013). Characterization of mycobacterial triacylglycerols and monomeromycolyl diacylglycerols from Mycobacterium smegmatis biofilm by electrospray ionization multiple-stage and high-resolution mass spectrometry. Anal. Bioanal. Chem..

[23] Davies E (2010). Dynamics of water in agar gels studied using low and high resolution 1H NMR spectroscopy. Int J. Food Sci. Technol..

[24] Barabesi C (2007). Bacillus subtilis gene cluster involved in calcium carbonate biomineralization. J. Bacteriol..

[25] Curthoys NP, Watford M (1995). Regulation of glutaminase activity and glutamine metabolism. Annu. Rev. Nutr..

