# Supplementary material for: Micro-CT X-ray imaging exposes structured diffusion barriers within biofilms
Source: NPJ Biofilms Microbiomes. 2018 Apr 17;4:8. doi: 10.1038/s41522-018-0051-8 (PMC5904145; doi:10.1038/s41522-018-0051-8)
Supplement: Supplementary file 1 — Supporting Material [file 41522_2018_51_MOESM1_ESM.pdf]

Micro-CT X-ray Imaging Exposes Structured Diffusion Barriers within Biofilms

Supplementary Information

SI File content:

1. Supplementary Movie Legends
2. Supplementary Figures and Figure Legends
3. Supplementary Materials and Methods
4. Supplementary References

1. Supplementary Movie Legends

Supplementary Movie 1: *B. subtilis* colony (24H, 20X magnification)

Supplementary Movie 2: *B. subtilis* colony (Day 3, 20X magnification)

Supplementary Movie 3: *B. subtilis* colony (Day 6, 10X magnification)

Supplementary Movie 4: *M. smegmatis* colony (Day 3, 10X magnification)

3D structure of biofilm colonies. *B. subtilis* and *M. smegmatis* were grown on B4 agar plates supplemented with calcium acetate as indicated in the main text. The intact colonies were visualized under X-ray, and the obtained 2D images were used to generate high-resolution 3D image (see Supporting Materials and Methods for details). Rotation of 360° was taken around an axis perpendicular to the biofilm surface.

2. Supplementary Figures and Figure Legends

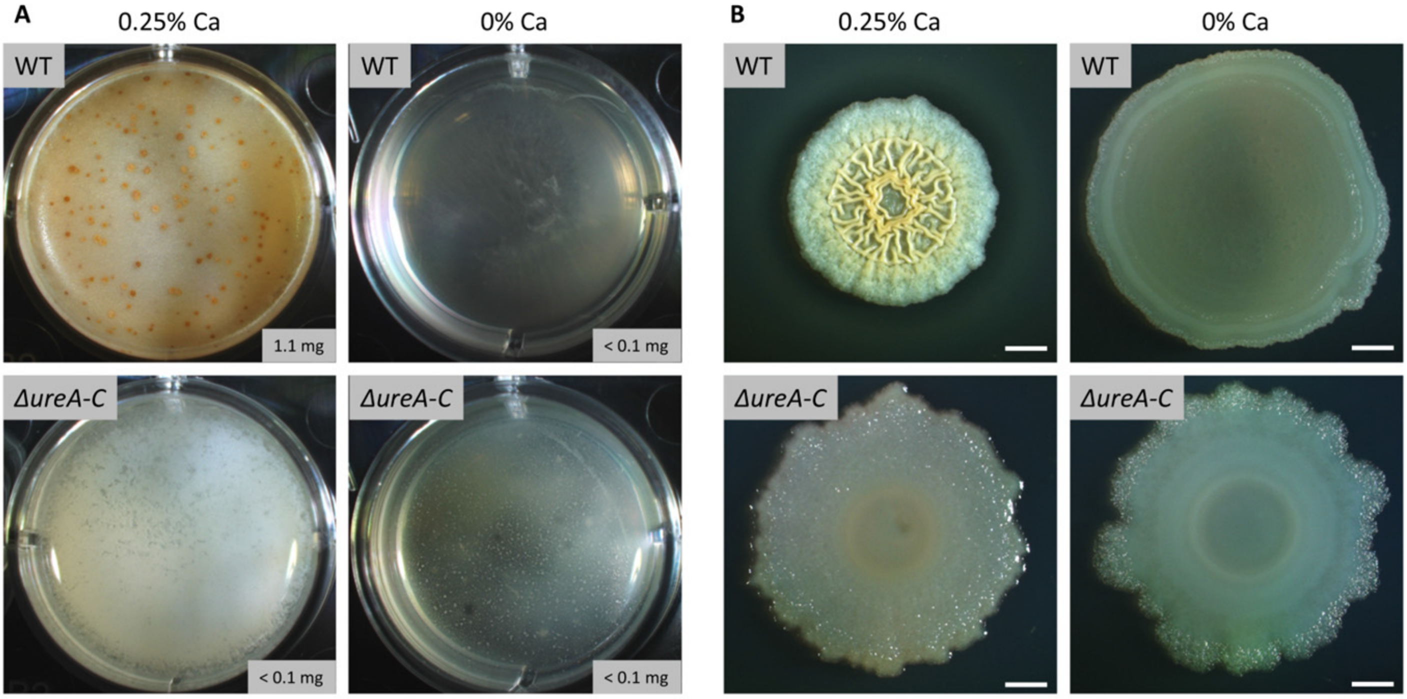

Supplementary Figure 1

B. subtilis cells (upper panel – wild type, lower panel - Δ*ureA-C*) were grown in: **A.** Liquid B4 without (-Ca) or with 0.25% calcium acetate. Pictures (top view of the wells) were taken after 6 days, and the weight of the mineral (insert) was determined after all organic material was removed by bleaching. Scale bar – 2 mm. B. B4 agar without (-Ca) or with 0.25% calcium acetate. Pictures of colonies were taken at day 3. Scale bar – 2 mm.

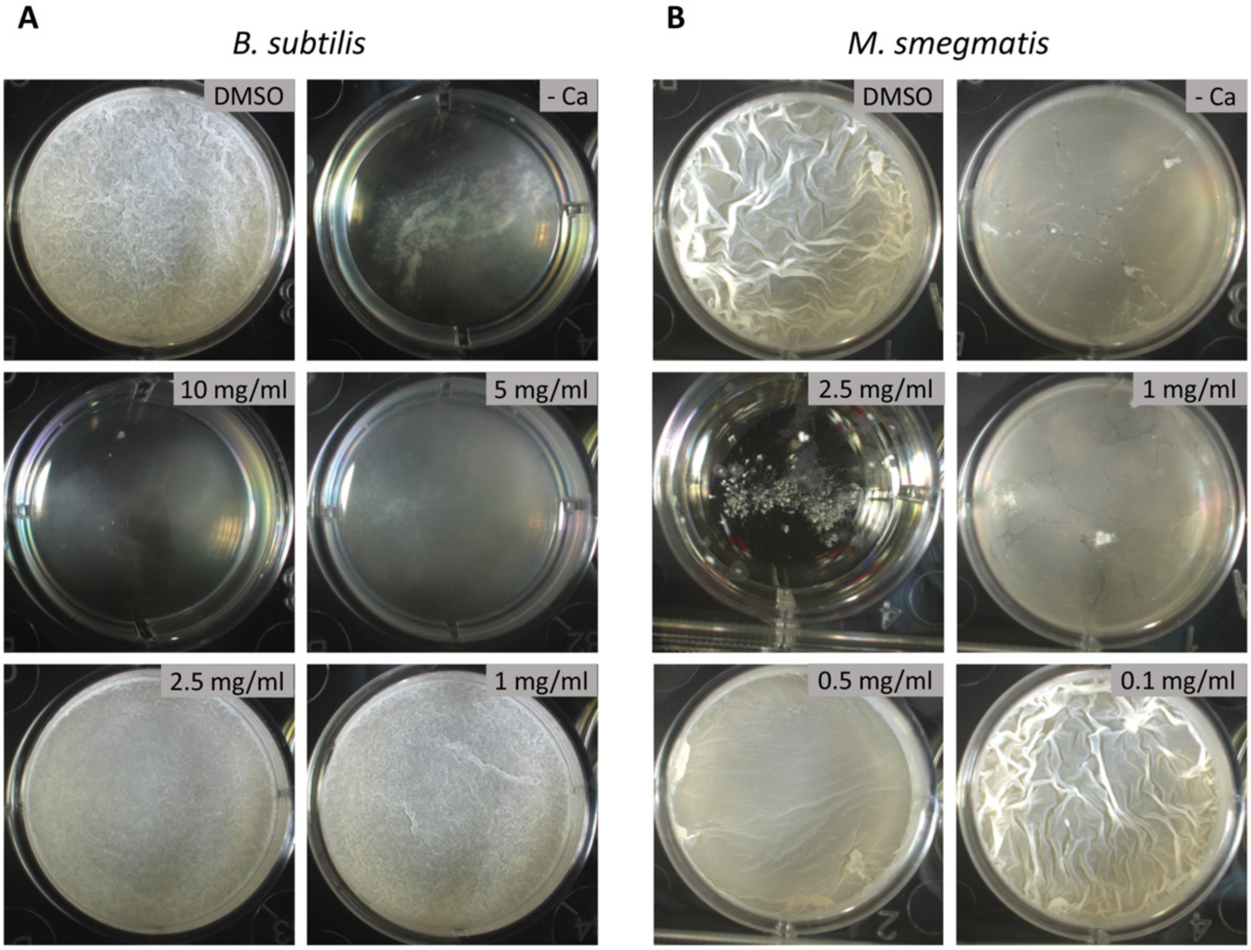

Supplementary Figure 2

A. *B. subtilis* and **B.** *M. smegmatis* cells were grown in liquid B4 without or with calcium acetate (0.25% and 0.025% respectively), supplemented with AHA at indicated concentrations. Pictures (top view of the wells) were taken after robust pellicles formed in the control – at 3 days for *B. subtilis* and at 4 days for *M. smegmatis*.

A

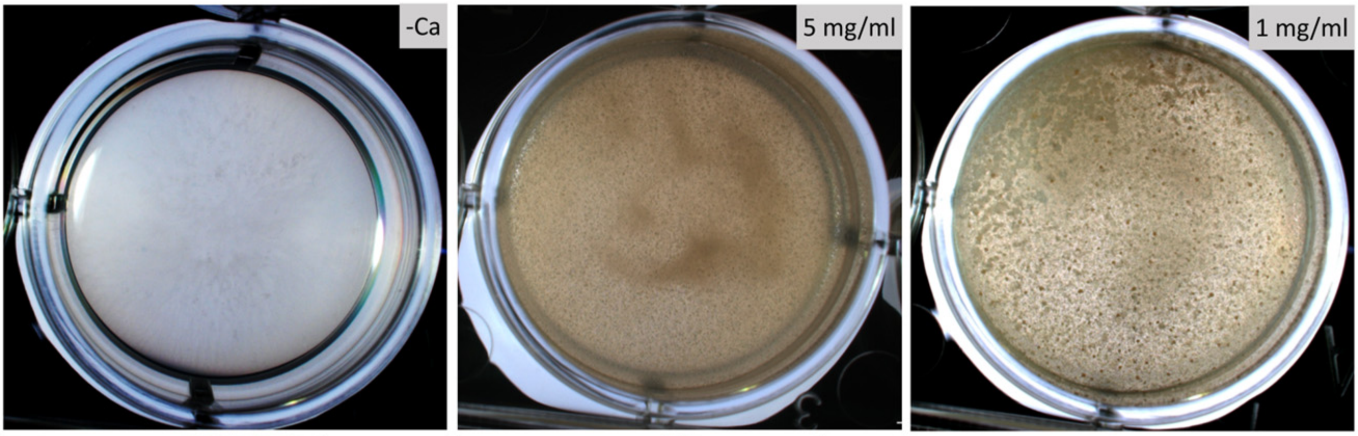

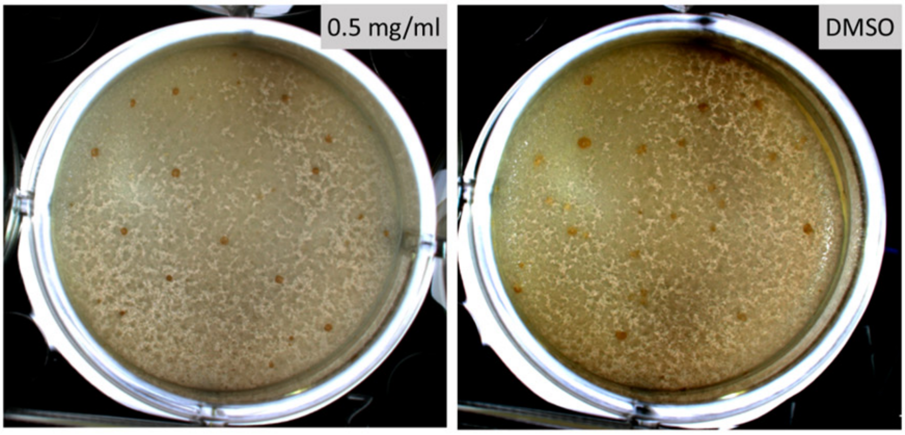

B

Treatment	Mineral Weight (mg)
- Ca	<0.1
AHA 5 mg/ml	<0.1
AHA 1 mg/ml	1.77 ± 0.15
AHA 0.5 mg/ml	1.57 ± 0.58
DMSO	1.85 ± 0.25

Supplementary Figure 3

B. subtilis cells were grown in liquid B4 without (-Ca) or with 0.25% calcium acetate, supplemented with AHA at indicated concentrations. Pictures (top view of the wells) were taken after 6 days, and the weight of the mineral was determined after all organic material was removed by bleaching.

3. Supplementary Materials and Methods

Strains and Media. *B. subtilis* NCIB 3610 (Branda et al., 2001) and *M. smegmatis* MC2155 (kindly provided by Dr. Eyal Gur) were grown at 30°C in B4 medium (0.4% yeast extract, 05% glucose) ((Barabesi et al., 2007)), supplemented with calcium acetate as indicated. Acetohydroxamic acid (AHA) was purchased from Merck (Cat. Num. 159034). FilmTracer™ Calcein Green Biofilm Stain (Cat. Num. F10322) was purchased from ThermoFisher Scientific.

Growth Analysis. To determine growth kinetics of planktonic bacteria, the cultures were grown for 15–30 h at 30°C in a plate reader (BioTek, Winooski, VT, USA), and the optical density at 600 nm (OD600) were measured every 20 min. The data shown in the figures are averages for 5-6 wells from a single representative experiment out of three.

Micro-CT X-ray analysis. Images of indicated magnification were taken using a Zeiss micro XCT 400 instrument (Pleasanton, CA, USA). Tomography was carried out using a micro-focused source set at 20 kV and 100 μA. 1200 separate 2D images were taken with a pixel size of 0.87 mm over 1800, exposure time of 30 sec. The volume was reconstructed from the complete set of images. Raw data were reconstructed with Zeiss software (Zeiss) that uses a filtered back-projection algorithm. To create the videos, 3D volume rendering (maximum intensity projection) was carried out with Avizo software (VSG, Hillsboro, OR, USA).

Imaging. All images were taken using a Nikon D3 camera or a Stereo Discovery V20" microscope (Tochigi, Japan) with objectives Plan Apo S ×0.5 FWD 134 mm or Apo S ×1.0 FWD 60 mm (Zeiss, Goettingen, Germany) attached to a high-resolution microscopy Axiocam camera. Data were created and processed using Axiovision suite software (Zeiss). To visualize colony cross-sections, after incubation with FITC, a warm low-melt agar was poured over the colony, allowed to solidify at RT, slices were made with a razor blade and immediately visualized. All experiments were repeated at least 3 times, in technical triplicates, with similar results.

Flow Cytometry. To determine the intracellular calcium levels, 3 day-old colonies were collected, sonicated gently to remove extracellular matrix and incubated for 1 h with FilmTracer™ Calcein Green Biofilm Stain prepared and diluted according to manufacturers' instructions. Next, cells were washed 3 times in PBS and sorted. Data was acquired with SORP-LSRII flow cytometer (BD Biosciences) and

analyzed with BD FACSDIVA™ software. The experiment was repeated 3 times, in technical duplicates, with similar results.

Analysis of the weight of the minerals. (A) Thermogravimetric analysis (TGA) of the *B. subtilis* colonies was performed as described previously (Levi-Kalisman et al., 2000) for at-least three colonies materials combined for each time point. The weight loss associated with the calcite relates to the temperature range 650–800 C°. Results are an average of at-least three independent experiments. (B) Weight of mineral in pellicle was determined as described by (Mahamid et al., 2008) et al., with some modifications: pellicle samples were slightly bleached with 3% sodium hypochlorite for 1 min to remove organic matter, washed twice with Milli-Q water (Merck KGaA, Darmstadt, Germany) and dehydrated in acetone. The experiment was repeated 3 times, in technical triplicates, with similar results.

4. Supplementary References

Barabesi, C., Galizzi, A., Mastromei, G., Rossi, M., Tamburini, E., and Perito, B. (2007). Bacillus subtilis gene cluster involved in calcium carbonate biomineralization. Journal of bacteriology *189*, 228-235.

Branda, S.S., Gonzalez-Pastor, J.E., Ben-Yehuda, S., Losick, R., and Kolter, R. (2001). Fruiting body formation by Bacillus subtilis. Proceedings of the National Academy of Sciences of the United States of America *98*, 11621-11626.

Levi-Kalisman, Y., Raz, S., Weiner, S., Addadi, L., and Sagi, I. (2000). X-Ray absorption spectroscopy studies on the structure of a biogenic "amorphous" calcium carbonate phase. J Chem Soc Dalton, 3977-3982.

Mahamid, J., Sharir, A., Addadi, L., and Weiner, S. (2008). Amorphous calcium phosphate is a major component of the forming fin bones of zebrafish: Indications for an amorphous precursor phase. Proceedings of the National Academy of Sciences of the United States of America *105*, 12748-12753.
